# A roadmap to integrating resilience into the practice of coral reef restoration

**DOI:** 10.1111/gcb.16212

**Published:** 2022-05-19

**Authors:** Elizabeth C. Shaver, Elizabeth McLeod, Margaux Y. Hein, Stephen R. Palumbi, Kate Quigley, Tali Vardi, Peter J. Mumby, David Smith, Phanor Montoya‐Maya, Erinn M. Muller, Anastazia T. Banaszak, Ian M. McLeod, David Wachenfeld

**Affiliations:** ^1^ The Nature Conservancy Arlington Virginia USA; ^2^ Marine Ecosystem Restoration Research and Consulting Monaco Monaco; ^3^ Stanford University Pacific Grove California USA; ^4^ Minderoo Foundation Perth Western Australia Australia; ^5^ ECS for NOAA Fisheries Office of Science & Technology Silver Spring Maryland USA; ^6^ Marine Spatial Ecology Lab, School of Biological Sciences, University of Queensland St Lucia Queensland Australia; ^7^ Coral Reef Research Unit School of Life Sciences Essex UK; ^8^ Mars Incorporated London UK; ^9^ Corales de Paz Cali Colombia; ^10^ Mote Marine Laboratory Sarasota Florida USA; ^11^ Universidad Nacional Autónoma de México Puerto Morelos Quintana Roo Mexico; ^12^ TropWATER, The Centre for Tropical Water and Aquatic Ecosystem Research, James Cook University Townsville Queensland Australia; ^13^ Great Barrier Reef Marine Park Authority Townsville Queensland Australia

**Keywords:** climate change adaptation, coral bleaching, coral reefs, resilience, resilience‐based management, restoration

## Abstract

Recent warm temperatures driven by climate change have caused mass coral bleaching and mortality across the world, prompting managers, policymakers, and conservation practitioners to embrace restoration as a strategy to sustain coral reefs. Despite a proliferation of new coral reef restoration efforts globally and increasing scientific recognition and research on interventions aimed at supporting reef resilience to climate impacts, few restoration programs are currently incorporating climate change and resilience in project design. As climate change will continue to degrade coral reefs for decades to come, guidance is needed to support managers and restoration practitioners to conduct restoration that promotes resilience through enhanced coral reef recovery, resistance, and adaptation. Here, we address this critical implementation gap by providing recommendations that integrate resilience principles into restoration design and practice, including for project planning and design, coral selection, site selection, and broader ecosystem context. We also discuss future opportunities to improve restoration methods to support enhanced outcomes for coral reefs in response to climate change. As coral reefs are one of the most vulnerable ecosystems to climate change, interventions that enhance reef resilience will help to ensure restoration efforts have a greater chance of success in a warming world. They are also more likely to provide essential contributions to global targets to protect natural biodiversity and the human communities that rely on reefs.

## Graphical abstract

Coral reefs are one of the most vulnerable ecosystems to climate change, and scientists have increasingly called for restoration interventions to be used to increase coral reef resilience to climate change. However, recent global reviews highlight a critical gap in implementation in the design and practice of restoration programs. We review the current literature and provide recommendations that integrate resilience principles into restoration design and practice for project planning and design, coral selection, site selection, and broader ecosystem context. We also discuss future opportunities to improve restoration methods to support enhanced outcomes for coral reefs in response to climate change.

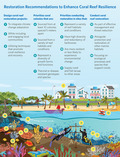

## 1 INTRODUCTION

The future of coral reefs is dependent on the rapid reduction of global greenhouse gas emissions and actions that enhance reef resilience to climate change (Kleypas et al., 2021). Across the globe, coral reefs are degrading due to human‐derived local threats (e.g., changes in land and sea use, pollution, overfishing) and anthropogenic climate change such as ocean warming and acidification (Cheal et al., 2017; Hughes et al., 2017; Shantz et al., 2020; Wear & Thurber, 2015). Recently, severe thermal stress events have caused over 70% of the world's reefs to suffer consecutive or prolonged bleaching events resulting in widespread losses of living corals (Eakin et al., 2019). For example, 14% of the world's coral reefs were lost in the decade from 2009 to 2018, due in part to successive and severe bleaching events from 2014 to 2017 that caused up to 95% coral mortality in some areas in the eastern Pacific (Brainard et al., 2018; Souter et al., 2021; Vargas‐Angel et al., 2019). Unless urgent action is taken to keep global mean temperatures from increasing beyond 1–1.5°C, most of the world's reefs are predicted to experience frequent bleaching, threatening the future of coral reefs and the human communities that depend on them (IPCC, 2022). In response, coral reef managers globally are increasingly turning to restoration to slow coral loss, rescue endangered species, and accelerate reef recovery processes (Boström‐Einarsson et al., 2020).

Ecological restoration is generally defined as the process of “assisting the recovery of an ecosystem that has been degraded, damaged, or destroyed” (Society for Ecological Restoration, 2004). However, this definition, along with current principles of ecological restoration, implies that the causes of ecosystem degradation and loss can be removed (Gann et al., 2019). While in local‐scale contexts this may be true for coral reefs (e.g., removal of blast fishing, herbivore overfishing, or wastewater pollution), global‐scale climate processes will likely continue to pose a significant threat to reefs for decades even if current targets for greenhouse gas emissions are met (IPCC, 2021). Thus, many scientists and governments now see restoration as a necessary management intervention to maintain coral reef ecosystem processes, functions, and services through the next few decades of climate change (Bay et al., 2019; Hein et al., 2021; Kleypas et al., 2021; Knowlton et al., 2021; Vardi et al., 2020).

Restoration also has been identified as a key component in resilience‐based management for coral reefs (Anthony et al., 2015; Knowlton et al., 2021; Mcleod et al., 2019). Resilience‐based management (RBM) focuses on prioritizing and implementing management actions to enhance reef resilience using knowledge of current and future threats (Mcleod et al., 2019). Underlying RBM is the theory of resilience, defined as the ability of a system to maintain key functions and processes in the face of stress by resisting, recovering, and/or adapting to change (Folke et al., 2010). More recently, resilience has been expanded to describe coupled social–ecological systems that can persist and transform to change (Keck & Sakdapolrak, 2013), where social resilience includes the ability of individuals, organizations, or communities to tolerate, absorb, and adapt to disturbances linked to changing environmental conditions and losses in resources (Keck & Sakdapolrak, 2013). Within the context of coral reefs, resilience refers to reef ecosystems that are less likely to be driven into persistent depauperate (e.g., algal‐dominated) states through: (1) resistance, where negative responses of corals to disturbances are reduced, limiting ecosystem change (e.g., less bleaching or less coral cover loss during warm temperature events); (2) recovery, where reef ecosystems more readily return to a predisturbance state (e.g., through rapid coral growth and coral recruitment); and (3) adaptation, where reef ecosystems are altered in response to changing conditions but continue to function and provide ecosystem services (e.g., due to changes in the dominance of coral species or taxa over time).

Research into emerging restoration techniques also increasingly focuses on improving coral or reef ecosystem resilience (Anthony et al., 2017; Van Oppen et al., 2015, 2017). Both the US National Academy of Sciences and Medicine (NASEM) and Australia's Reef Restoration and Adaptation Program (RRAP) conducted recent large‐scale reviews to identify current and future interventions with the potential to promote resilience and assess their potential feasibility, scale, and risks (Bay et al., 2019; NASEM, 2019). Most recently, Suggett and van Oppen (2022) illustrate how these novel approaches (e.g., probiotics, selective breeding, assisted evolution, bio‐banking) can be used in the asexual–sexual coral life cycle to improve restoration success. Meanwhile, other recent publications have provided broadscale guidance for coral reef restoration (e.g., Hein et al., 2021; Quigley et al., 2022; Shaver et al., 2020), which include recommendations aligning with resilience theory (e.g., maximizing biodiversity and promoting connectivity: Nyström et al., 2008), but do not directly relate restoration practice to resilience.

Despite clear recognition in the scientific literature for resilience and climate‐focused restoration techniques, there remains a critical gap in implementation. For example, in a global review of over 350 reef restoration projects up to 2018, only five projects included the word “climate” in the project description or goals (Boström‐Einarsson et al., 2020). While resilience calls for increasing diversity (i.e., species, habitat) to spread the risk of loss from a disturbance event (McLeod et al., 2012; Mcleod et al., 2019; Nyström et al., 2008), nearly a third (28%) of projects in this review focused on just one coral species, the majority of which (59%) were branching corals that are generally less resilient to climate change‐related bleaching (Boström‐Einarsson et al., 2020; Loya et al., 2001; van Woesik et al., 2011). Coral reef restoration efforts are also often led by local community‐based organizations or management agencies that may not have scientists on staff or have access to scientific publications. Thus, for most practitioners, it is likely not clear how restoration should be conducted to promote reef resilience, and indeed no current resources exist that synthesize the science to describe approaches that are currently available for resilience‐based coral reef restoration design.

**FIGURE 1:**
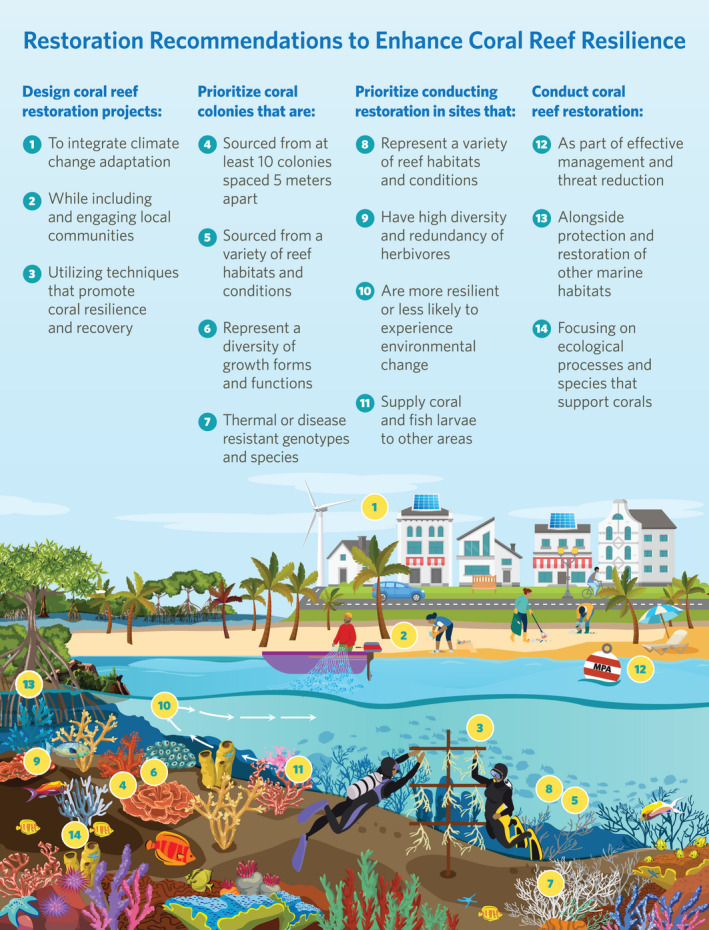
Illustration of recommendations for enhancing coral reef resilience through restoration design and implementation

Here, we address this implementation gap by providing guidance for how coral restoration practitioners, managers, and communities can incorporate resilience principles and climate considerations into coral reef restoration practice. We organize our guidance into four categories: (1) project planning and design, (2) coral selection, (3) site selection, and (4) broader ecosystem context (Table 1, Figure 1). As scientists warn that coral reefs may be the first ecosystem to be lost to climate change (Kleypas et al., 2021), we present these recommendations with the goal of supporting and catalyzing the coral reef restoration community to shift toward more climate‐smart and resilience‐focused coral reef restoration.

**TABLE 1:** Recommendations for incorporating resilience principles and considerations into the design and implementation of coral reef restoration. “Operational status” refers to the ability of practitioners to implement the recommendation in restoration programs at this current time (scale: 1 = operational with *many* challenges; 2 = operational with *some* challenges; 3 = operational with *few* challenges), determined by averaging the ratings of coral reef experts (*n* = 9). “Implementation needs or dependencies” includes any data, information, or processes that are to be likely required by restoration practitioners to implement the recommendation

Recommendation	Operational status (1–3)	Implementation needs or dependencies	References
**Project planning and design**			
Integrate environmental change and climate adaptation into restoration planning	2	Climate adaptation design tools Reef resilience assessments Climate vulnerability assessments Models of past and future local and global threats downscaled to smaller spatial scales	West et al., 2018; Shaver et al., 2020
Include local communities and traditional and local knowledge in restoration projects to support social–ecological resilience	3	Identification of key stakeholders Informational stakeholder meetings Stakeholder education and outreach Early engagement in project planning Socioeconomic data including cultural dynamics	Kittinger et al., 2016; Fox & Cundill, 2018; Hein et al., 2019
Utilize techniques that promote genetic diversity, increased thermal tolerance, and rapid coral recovery	2	Funding for advanced techniques Technical capacity with expertise Coral genotyping and inventories Monitoring donor and nursery corals for thermal tolerance	Bay et al., 2019; NASEM, 2019; Suggett & van Oppen, 2022
**Coral selection**			
Source corals from a diversity of genotypes by collecting corals from at least 10 unique genets spaced no less than 5 meters apart	3	Donor coral genotyping and inventories Donor collections at distance Field training and education	Shearer et al., 2009; Baums et al., 2019
Source corals from a variety of reef habitats including diverse environments and conditions	3	Habitat mapping across larger reef system Ecological and environmental coral reef data Incorporation of traditional and local knowledge Corals at multiple donor sites Monitoring of success based on source and outplanting location	McLeod et al., 2009; Torda & Quigley, 2021
Restore a diversity of coral phenotypes, growth forms, and functional roles	2	Funding and technical capacity for multiple propagation techniques Access to diverse brood stock at donor sites Assessment of local coral assemblages, phenotypes, and functional roles	Nyström et al., 2008; Veron, 2011
Use thermal or disease‐resistant species and genotypes, but when not known increase genotypic and morphological diversity to incorporate varying tolerances and promote redundancy	2	Monitoring of donor and nursery colonies Genetic sequencing Funding and technical capacity for techniques Access and mapping of diverse brood stock at donor sites	Morikawa & Palumbi, 2019; Quigley et al., 2020; Barott et al., 2021
**Site selection**			
Conduct restoration in multiple sites that represent a variety of reef habitats, such as depths, oceanographic conditions, and thermal regimes	2	Monitoring of species distribution, cover, health status across larger reef system Capacity, logistical, and financial resources Connectivity and ocean circulation data or modeling	Elmqvist et al., 2003; Nyström et al., 2008; McLeod et al., 2009
Select sites with high diversity and functional redundancy of reef herbivores	2	Surveys of herbivore diversity and abundance Effective herbivore management Technical expertise for herbivore surveys	Elmqvist et al., 2003; Burkepile & Hay, 2008
Conduct restoration in areas that show higher resilience to, or are less likely to experience, environmental or climate change impacts	2	Reef resilience assessments Reef monitoring during bleaching/disease events Models of past and future local and global threats downscaled to smaller spatial scales Incorporation of traditional and local knowledge Funding/technical capacity for surveys or modeling	McLeod et al., 2009; Oliver & Palumbi, 2011; McLeod et al., 2012; Chollett et al., 2022
Prioritize sites that provide high larval output to other areas, accommodating dispersal distances of coral species of interest	2	Hydrodynamic connectivity models downscaled to smaller spatial scales Monitoring of recruitment across reef system Incorporation of traditional and local knowledge Larval characteristics data for target coral species	Schill et al., 2015; Magris et al., 2016; Hock et al., 2017; Quigley et al., 2019; Mumby, Mason, & Hock, 2021
**Broader ecosystem context**			
Ensure restoration is integrated within a broader resilience‐based management strategy, focused on reducing local threats to reefs prior to restoration	2	Collaborations with reef managers and stakeholders Management and conservation planning Assessment of local threats and related management authorities Management intervention monitoring Incorporation of traditional and local knowledge Political, social, and economic support	Mcleod et al., 2019; Shaver et al., 2020; Hein et al., 2021
Restore or protect multiple ecologically connected marine habitats and ecosystems	2	Effective landscape‐scale management Collaborations with practitioners or management authorities from other habitats Knowledge of restoration in other habitats Ecological and oceanographic connectivity modeling across ecosystems Incorporation of traditional and local knowledge Funding and technical capacity for techniques	Milbrandt et al., 2015; van de Koppel et al., 2015
Restore processes and populations of non‐coral species that support coral reef functional processes and recovery	1	Ecological assessment of reef species and functional roles Pilot research on interventions Funding and technical capacity for techniques	Shaver & Silliman, 2017; Ladd et al., 2018

## 2 RECOMMENDATIONS FOR RESTORING REEFS IN A CHANGING CLIMATE

### 2.1 Project planning and design

An important principle in ecological restoration includes the consideration of natural variation and anticipated future environmental change when identifying restoration targets (Gann et al., 2019). Despite the systematic incorporation of climate change impacts into marine spatial planning (Beyer et al., 2018; McLeod et al., 2012), marine reserve design (Mumby et al., 2011), and watershed management (Gibbs et al., 2021), only recently has guidance for coral reef restoration included climate change data in project planning and design (Shaver et al., 2020). As global warming will continue for decades regardless of near‐term reductions in greenhouse gas emissions, current coral reef restoration projects must be designed for predicted future climate change impacts, including how climate change could affect restored coral species, methods used (e.g., storm impacts on artificial reefs), and the location of efforts. The use of climate change adaptation tools, developed for designing other reef management strategies (West et al., 2018), can also be used for coral reef restoration planning (e.g., Shaver et al., 2020). Other tools that examine the role of climate change on local social and ecological conditions or resilience at local sites (i.e., climate vulnerability assessments, reef resilience assessments) also can help to ensure that climate change considerations are embedded in early project planning and design (Table 1).

Considerations of social resilience for reef‐dependent communities should also be included in restoration planning and design, such as how restoration programs can provide increased food security (e.g., improved fisheries), infrastructure security (e.g., improved coastal protection), or livelihoods (e.g., eco‐tourism opportunities) (Table 1). A new priority for resilience‐based coral reef management includes strategies that strengthen social adaptive capacity (a core component of social vulnerability and resilience) that allows communities to prepare for, cope with, and adapt to reef change (Mcleod et al., 2019). Strategies can include supporting economic diversity and livelihood opportunities, supporting the leadership of Indigenous peoples and local communities, and incorporating traditional knowledge and local values and perspectives into projects (Berkes & Seixas, 2005; Cinner et al., 2012; Marshall et al., 2007; McClanahan et al., 2008). Restoration is particularly suitable for building social adaptive capacity, especially when considered in project design, and many programs are increasingly including Indigenous peoples, the eco‐tourism sector, and local fishers into their programs.

Partnerships between local communities, Indigenous groups, management agencies, and scientists enable the integration of traditional and local ecological knowledge with climate projection data for project planning such as the selection of restoration sites (Gann et al., 2019; Hein et al., 2021). Additionally, strong stakeholder participation in restoration planning or project implementation could build community buy‐in and support for the project, drive behavioral change, increase education to address other reef threats, or support “reef‐positive” livelihood opportunities (Fox & Cundill, 2018; Hein et al., 2019; Kittinger et al., 2016), thereby reducing community vulnerability to reef loss and supporting social and ecological resilience as well as overall project success.

Several restoration techniques that support improved coral resistance, recovery, or adaptation should also be considered during restoration planning and design to support climate and resilience‐focused restoration projects (Table 1). Scientists at Mote Marine Laboratory in Florida, for example, are using techniques with a focus on resilience including selective breeding (i.e., selecting corals with phenotypic traits related to stress tolerance for breeding) to identify a variety of coral genotypes and species that appear to be resilient to temperature stress, ocean acidification, and disease. These techniques are integrated with other methods that promote genetic diversity of corals through sexual reproduction (e.g., larval propagation) to enhance the potential for coral adaptation to climate change and increase the number of corals outplanted with tolerance to these stressors. Balancing selective breeding with natural sexual reproduction should help ensure that the genetic integrity of outplanted offspring is not eroded or that potential trade‐offs in fitness traits are minimized or controlled during breeding. In another example, Australia's Reef Restoration and Adaptation Program is researching a range of interventions to help sustain coral reefs in a changing climate (Bay et al., 2019), including methods that support resistance and recovery (e.g., enhancing larval settlement and reef accretion, stabilizing unconsolidated reef substrate, and symbiont manipulation to develop climate resilient stock) to boost reef resilience following disturbances (Bay et al., 2019; Ceccarelli et al., 2020).

Although many emerging techniques are still being developed globally, a key priority moving forward is to develop restoration interventions that are affordable and accessible to practitioners across all reef regions. For instance, larval‐based restoration can be an economical and effective option for increasing genetic diversity into localized, existing coral populations, and the Coralium Laboratory under the National Autonomous University of Mexico is currently focused on developing low‐cost field laboratories for larval propagation and coral husbandry. While mechanisms currently exist to support practitioners in incorporating resilience and climate change into their restoration designs, further research on processes for coral adaptation (e.g., coral upper thermal limits, heritability) and methods to support reef resilience (e.g., new interventions, predictive coral traits for resilience) will be critical to informing these efforts.

### 2.2 Coral selection

One of the most common approaches in coral reef restoration utilizes underwater nurseries to grow branching corals, such as the Caribbean staghorn coral *Acropora cervicornis* (Young et al., 2012). This single‐species approach to growing, propagating, and outplanting corals stemmed from work in Florida focused on repopulating *A. cervicornis*, a once dominant reef builder that is now critically endangered throughout the Caribbean (Aronson et al., 2008). Branching coral species like acroporids are commonly used in restoration because they can be easily fragmented and grow rapidly, allowing practitioners to experiment with coral propagation and nursery methods that are now foundational approaches to coral reef restoration. While there may be instances where a single‐species approach is appropriate based on a specific restoration goal (e.g., planting branching corals such as *Acropora palmata* on the reef crest to improve coastal protection services), in general scientists are raising the alarm that coral reef restoration practices must move from a focus on single species and coral outplanting to ecosystem‐wide approaches to ensure reef survival to climate change (Hein et al., 2021; Vardi et al., 2021). Indeed, restoration programs are increasingly incorporating multiple coral species and growth forms, though most efforts still center around coral outplanting (Boström‐Einarsson et al., 2020).

This shift to incorporate multiple coral species in reef restoration is essential as increased diversity and functional redundancy are core components of ecosystem resilience (Biggs et al., 2012; Elmqvist et al., 2003; Mcleod et al., 2019; Nyström et al., 2008). Specifically, diversity promotes a varied response to disturbance, potentially conferring increased resistance (e.g., less bleaching for some species or genotypes) and recovery (e.g., faster growth rates of some species) of the reef system to climate change impacts. Functional redundancy, where different species provide similar ecological functions (i.e., multiple branching coral species that provide habitat complexity to fish and invertebrates), allows the ecosystem to recover, adapt, and continue functioning after a disturbance even if one species is lost.

Practitioners should seek to incorporate diversity into their restoration programs through using different coral species and genotypes representing a variety of growth forms (and thus, ecological functions). To integrate diversity at the genetic level into restoration projects, corals should be sourced from a variety of habitats with diverse environmental conditions within species boundaries in the restoration region (Table 1). In less genotypically diverse habitats, practitioners should source corals of the same species from different populations to capture a range of phenotypic traits and genotypes, as genetic diversity can be highly variable across coral species and reef habitats (Shearer et al., 2009; Torda & Quigley, 2021). Current research using four Caribbean coral species suggests that collecting coral fragments from 10 to 35 genetically distinct donor colonies (i.e., “genets”) should capture the majority (50%–95%) of genetic diversity within a species (Shearer et al., 2009). Confirming unique genets by sequencing donor colonies is recommended when possible; however, collecting fragments from corals spaced at least 5 m apart (ideally larger, such as 50 m) and/or of various phenotypes will promote a diverse genetic composition if sequencing is not available or affordable (Baums et al., 2019) (Table 1). Because of differences in local contexts, however, when possible, practitioners should seek to identify the spatial variability of genotypes for target coral species in their location to determine the number of corals and spacing between donors required for collecting dis genotypes.

Corals with various morphologies (e.g., branching, massive, plating, foliose) fulfill a variety of functional roles within the reef habitat (Veron, 2011) and tend to have different tolerances to environmental stresses, due to factors such as size, shape, tissue thickness, energy allocation, and associations with algal symbionts with different thermal tolerances (Baker, 2003; Grottoli et al., 2014; van Woesik et al., 2011). For example, fast‐growing branching corals are in general less thermally tolerant than slow‐growing massive corals, likely because they have thinner tissues with less energy reserves than massive species (Loya et al., 2001; van Woesik et al., 2011). Restoration incorporating multiple species with different morphologies could lead to a diversity of responses to environmental conditions, thereby promoting reef resilience by buffering against widespread coral loss from a single event. While using thermally tolerant massive corals may enhance reef resistance to climate change events, fast‐growing branching corals will be useful for promoting rapid recovery after large‐scale disturbances. Thus, a mix of coral morphologies is key to promoting reef resilience, and future research should seek to identify and develop techniques that decrease the time needed to propagate a diversity of coral growth forms (Table 1).

Thermally‐resistant coral species should be included in restoration projects, such as those that have a history of surviving stress or are naturally acclimated to environmental extremes, to promote the mixing of heat tolerance genes within future generations (Gardner et al., 2019; Palumbi et al., 2014; Quigley et al., 2020) (Table 1). Heat tolerance appears to be at least partially heritable across multiple coral species and targeting resilient parents for sexual reproduction can lead to increased tolerance in offspring (Dixon et al., 2015; Dziedzic et al., 2019; Quigley et al., 2021). When bleaching‐susceptible species are critical for reef community recovery (e.g., acroporids in the Caribbean), integrating genotypes that are more heat tolerant into propagation is essential. Recent research shows that bleaching resistance of heat‐tolerant corals can be maintained within nurseries (Morikawa & Palumbi, 2019) and after direct transplantation (Barott et al., 2021). Thus, testing nursery‐reared corals for thermal tolerance and including heat‐tolerant corals in outplanting sites may help ensure enhanced population persistence of that species after warming events. However, whether adaptation will occur and spread through populations rapidly enough to keep pace with increasing temperatures remains less certain (Bay & Palumbi, 2017; Quigley et al., 2019). Similar approaches can be taken for identifying coral genotypes and species that are resistant to coral disease. Practitioners can identify resistant corals through low‐cost means, such as tracking genotypes in nurseries or conducting routine monitoring on reefs with tagged corals to identify differences in tolerances before and after disturbances events (i.e., susceptibility and severity of different corals before and after bleaching or disease events). Further research to develop affordable genotyping tools that can be used in the field will be critical to support practitioners in these efforts.

Importantly, there are possible trade‐offs between resilience traits (e.g., heat tolerance) and growth in corals (Cornwell et al., 2021), although some traits appear to be independent (e.g., heat stress and disease resistance: Muller et al., 2018). Resistance to heat stress and ocean acidification, for instance, have been positively associated for endangered *A. cervicornis* (Muller et al., 2021). Therefore, ensuring that a wide diversity of coral genotypes, species, and growth forms are used in restoration efforts is likely the best course of action until potential trade‐offs can be identified through additional research. Methods to enhance genetic variation will be needed in combination with outplanting diverse corals, including the integration of larvae from as many parental donors as possible or the use of as many heat‐resistant corals as possible in nurseries (Cornwell et al., 2021). Ultimately, practitioners should monitor different coral species, genotypes, growth forms, and sizes before and after disturbance events (both in the short and long‐term) to determine the best coral assemblage to use for their specific restoration goals and context.

### 2.3 Site selection

Site selection for restoration is another key area where resilience components should be factored into restoration design. For instance, models show that prioritizing habitat diversity can protect heat‐resistant coral populations and promote coral adaptation (Walsworth et al., 2019). Practitioners should seek to conduct restoration in sites that span a variety of reef types (i.e., fringing, barrier, and patch reefs) and conditions, including differences in depths, oceanographic features, and thermal regimes, with replication across site types whenever possible (Nyström et al., 2008; van Nes & Scheffer, 2005; Walsworth et al., 2019) (Table 1). Sites with high diversity and functional redundancy of herbivores (which reduce macroalgae and/or promote substrate conditioning for coral larval settlement) could also be used as site selection criteria (Burkepile & Hay, 2008; Elmqvist et al., 2003) to support increased coral recovery by keeping macroalgal cover in check.

To identify sites that have the highest potential for resilience, practitioners should work with marine managers or scientists to conduct resilience assessments to identify and prioritize locally resilient reefs for restoration outplanting (Shaver et al., 2020). Resilience assessments, for example, have been used since 2007 by reef managers and scientists in every coral reef region to identify reefs with a higher potential to survive future climate change and prioritize them for management actions (Mcleod et al., 2021). Yet, in a review of how resilience assessments have been used to inform reef management actions to date, only one project used resilience assessment results to identify and select sites for restoration (Mcleod et al., 2021). Resilience assessments provide critical information on underlying factors leading to higher or lower resilience in different sites (e.g., oceanographic features, water quality conditions, herbivory, and recruitment rates), and therefore can be used to enhance restoration outcomes by identifying resilient sites and informing management activities that should be conducted prior to restoration (Table 1).

To mitigate future risks to restoration brought about by changing environmental conditions, local and global knowledge of predicted climate impacts at potential restoration sites should also be incorporated. Ideally, climate change refuges that are the least at risk from future climate change would be identified and prioritized for restoration (see Chollett et al., 2022 and 50 Reefs, 50reefs.org). This could include areas that (1) are reliably cooled, (2) regularly experience high thermal variability or extreme conditions, (3) do not experience regular intense storm activity, or (4) are projected to be less impacted by future warming or acidification (Fine et al., 2013; McLeod et al., 2009, 2012; Oliver & Palumbi, 2011; Randall et al., 2020) (Table 1). Recent research on consecutive bleaching events on the Great Barrier Reef shows there is consistency in thermal regimes of reefs, suggesting the locations of refugia and hotspots can be robust and predictable (Cheung et al., 2021). Using data on thermal stress patterns (i.e., historical and projected sea surface temperatures) can help practitioners select restoration sites with a greater likelihood of success in a changing climate, as has been used in marine reserve design (Mumby et al., 2011) and most recently for coral reef restoration (Chollett et al., 2022). For practitioners in the Caribbean and Florida, this information is available for coral reefs down to the 1‐km scale through The Nature Conservancy's Caribbean Coral Climate Refugia Data Explorer (CoralRefugia.tnc.org). One potential low‐cost approach to identifying resilient reef sites includes rapid and standardized testing of coral thermal tolerance using portable devices (Oliver & Palumbi, 2011; Thomas et al., 2018). Voolstra et al. (2020) reengineered these as the Coral Bleaching Automated Stress System (CBASS), which tests the responses of small coral samples to acute thermal stress in the field. This system could also identify naturally heat‐resistant corals for use as donor colonies for restoration or direct transplantation. However, comparisons between ecologically relevant scenarios and portable stress‐test systems such as CBASS will also require further exploration.

Identifying larval connectivity patterns at potential restoration sites is also an important consideration for designing restoration to promote resilience (Table 1). The value of locally protected, thermally resilient reefs is enhanced when these corals act as sources of larvae to nearby areas (Hock et al., 2017; Mumby, Mason, & Hock, 2021; Mumby, Steneck, et al., 2021), spreading heat resilience traits. For instance, sites identified as thermal refugia may be capable of providing coral larvae to 58% of the Great Barrier Reef, highlighting the importance of restoring such sites to provide system‐wide reef resilience (Cheung et al., 2021). Restoration projects should be located both within and across reefs to accommodate different larval dispersal characteristics of key species of interest (e.g., considering species‐specific reproductive strategies and local oceanographic conditions; Magris et al., 2016) and different environmental regimes leading to variable conditions in local adaptation. When possible, larval connectivity studies overlaid with model predictions on future climate conditions should be used to prioritize sites for restoration based on the movement of coral larvae of known source and sink locations (see Chollett et al., 2022), as have been used to design marine protected area networks (Magris et al., 2016; Schill et al., 2015). For instance, sink reefs (e.g., that receive a large portion of larvae from other areas) in theory may be good candidates for donor coral collection because coral diversity may be higher in these sites. In contrast, source reefs (e.g., that export a large portion of larvae to other areas) may be good sites for outplanting because restored colonies in these areas could support higher recovery to nearby connected reefs. While methods do currently exist for use in restoration, more research and investment are needed to develop and make available predictive larval connectivity studies at local scales for use by restoration practitioner groups (e.g., Frys et al., 2020).

### 2.4 Broader ecosystem context

Coral reef restoration projects aimed at supporting resilience and climate adaptation of corals cannot be fully realized without considering the broader context within which reef ecosystems function, including connections between adjacent marine habitats and human populations. Ecological connections between ecosystems across the land and seascape are well known to affect reef resilience (e.g., Guannel et al., 2016; Mumby & Hastings, 2008). Recent research highlights how restoration outcomes are improved when multiple degraded and ecologically connected ecosystems are restored together (Milbrandt et al., 2015; van de Koppel et al., 2015) (Table 1). For example, intact mangroves and seagrasses may benefit nearby coral reef restoration efforts by improving water quality and alkalinity (e.g., Guannel et al., 2016; Manzello et al., 2012). Restoring mangroves may help to mitigate the effects of lost coral reef structural complexity on reef fish biomass and fisheries productivity, offsetting some of the impacts of climate change on neighboring reefs in terms of fish biodiversity (e.g., Rogers & Mumby, 2019). Likewise, restoring terrestrial forests and riparian vegetation could reduce sediment flow into adjacent coral reefs, supporting improved survivorship and fitness of coral outplants (e.g., Carlson et al., 2019).

An intact ecosystem that has redundancy and feedback systems in place is more likely to show increased resilience compared with single‐species monocultures (Downing & Leibold, 2010; Nyström et al., 2008; Vogel et al., 2012). Thus, as coral reef restoration projects mature and increase in scale, methods used should transition from a focus on single species and coral outplanting to approaches that improve ecological processes and functioning (Hein et al., 2021; Vardi et al., 2021). One way may be to incorporate non‐coral species, particularly those known to facilitate coral recovery, recruitment, and health (Ladd et al., 2018, Shaver & Silliman, 2017) (Table 1). For example, herbivores that graze algae and provide suitable substrate for coral settlement could potentially enhance the success of restoration projects (Ceccarelli et al., 2018; Spadaro & Butler IV, 2021; Williams, 2022). In Hawaii, the cultivation and transplantation of the urchin *Tripneustes gratilla*, in combination with manual removal methods, has been used by the Hawaii Division of Aquatic Resources (USA) to control invasive macroalgae and rehabilitate reefs (Conklin & Smith, 2005; Neilson et al., 2018). Herbivorous snails, used in co‐culture with ex situ sexually propagated coral recruits, were found to increase coral survival 23‐fold (Neil et al., 2021). In another example, encrusting sponges and coralline algae were investigated as natural mechanisms to secure coral rubble and promote recruitment on damaged reefs (Biggs, 2013). Conversely, practitioners may need to incorporate restoration interventions or designs that mitigate the impacts of non‐coral species that reduce coral recovery potential, such as corallivores like Crown‐of‐Thorns Starfish (COTS) or *Drupella* snails. However, more research on processes and species that promote coral health and resilience, as well as interventions and techniques for restoring non‐coral species, is required for practitioners to utilize interspecific relationships to promote coral reefs through restoration.

Landscape‐level connections to local human populations are also critical considerations for coral reef restoration designs to support resilience. For instance, anthropogenic stressors, particularly nutrients and other pollution from terrestrial sources, are known to reduce reef resilience (Carilli et al., 2009; Donovan et al., 2020; Vega Thurber et al., 2014), and management actions to mitigate such local stressors can improve resilience (Mumby, Steneck, et al., 2021; Shaver et al., 2018). To ensure local threats are mitigated in existing or potential restoration areas, restoration should be embedded within a broader management framework and deployed in areas where local threats can be controlled (Mcleod et al., 2019) (Table 1). This could include marine protected areas, other effective area‐based conservation measures, or coastal zone management areas where the impacts of overfishing, tourism, coastal development, or marine vessels are reduced. Efforts to manage or restore watersheds to reduce nutrient pollution, sedimentation, and sewage should be undertaken alongside, but ideally before, restoration begins, to improve coral outplant success (Hein et al., 2021; Shaver et al., 2020). These efforts should also ensure consideration of the social–ecological context in reef management to strengthen social adaptive capacity, resilience, and thus overall compliance with management and restoration actions (McLeod et al., 2012).

## 3 FUTURE DIRECTIONS

Building resilience into coral reef restoration will require new partnerships and the testing and integration of novel biological, ecological, social, and oceanographic methods that specifically target and enhance the mechanisms of coral reef recovery, resistance, and adaptation to local and global disturbances. For example, mechanisms that improve coral recruitment (i.e., survivorship rates of recruitment) could be an important research frontier to enhance coral population recovery after disturbance. Examples include innovations in new materials (e.g., hydrogels) to protect corals in vulnerable early life stages and increase survivorship of coral recruits (Randall et al., 2019), the incorporation of crustose coralline algae and biofilms in restoration projects (Heyward & Negri, 1999), or the use of acoustic playback of a healthy reef to enhance coral settlement in degraded sites (Gordon et al., 2019; Lillis et al., 2016).

The number of coral fragments available for outplanting is currently one of the most significant bottlenecks to scaling up restoration, limiting the spatial scale of efforts as well as the diversity of species, growth forms, and genets critical for enhancing ecological resilience. Coral reef restoration could utilize processes and lessons learned from current practices in terrestrial habitats, for example where terrestrial nurseries (managed as separate entities from restoration projects) provide diverse species for restoration practitioners that are acclimated for distinct microhabitats. For example, a regional coral nursery might stock coral fragments of different growth forms and genotypes suited to different environmental conditions (e.g., flow, depth, and thermal conditions) for a range of restoration projects in the area. Such facilities have already been constructed in some reef environments such as hatcheries for giant clams, turbo snails, or other species (e.g., Mies et al., 2017) but will require more research and changes in policy to enable the movement of coral specimens across larger geographic areas. The field of coral reef restoration can also learn from the aquaculture industry in terms of mass‐scale culture. Australia's Reef Restoration and Adaptation Program, for instance, is focusing on the use of automation to optimize coral rearing and deployment while improving outplant survival rates. Such innovations will be key for enabling practitioners to produce the diversity and abundance of corals needed to restore reefs at scales large enough to combat reef losses.

The future success of coral reef restoration efforts through the next century of climate change will require greater collaboration between scientists, practitioners, managers, Indigenous Peoples, and public and private sector investors to ensure that projects meet local needs, benefits are equitably distributed, and information is applicable to local restoration efforts. Additionally, restoration practitioners should be included in the research design phase for new interventions and written into grant proposals to ensure new technologies are trialed and ultimately usable and affordable to support broad‐scale application. More emphasis should be placed on trainings and support to equip local practitioners to utilize new interventions and deliver projects at scale and with maximum resilience of reef ecosystems. Groups that support knowledge exchange, such as the Coral Restoration Consortium, the Nature Conservancy's Reef Resilience Network, and the International Coral Reef Initiative, provide important opportunities for sharing best practices in coral reef restoration to support the scaling of effective approaches.

### 3.1 CONCLUSION

As the UN Decade on Ecosystem Restoration (2021–2030) begins and nations seek to meet ambitious conservation and biodiversity targets, it is necessary to conduct restoration as part of broader resilience‐based management of coral reefs and incorporate resilience principles and climate change adaptation into restoration practice. The recommendations presented here provide guidance to help the coral reef restoration community enhance reef resilience to climate change and other reef threats (e.g., disease) (Figure 1). Recommendations are in line with key principles for the practice of ecological restoration that guide all practitioners involved in restoring degraded habitats (i.e., Gann et al., 2019), suggesting this guidance could be applied to efforts in other terrestrial, freshwater, coastal, or marine ecosystems. Ideally, restoration projects would implement most or all of these recommendations (Figure 1); however, it is likely that projects will need to prioritize recommendations depending on their local context and needs, including logistical constraints or different stakeholder objectives. Potential strategies to prioritize and select recommendations include multicriteria analysis, deliberative democracy, or codesign approaches, which would allow organizations or institutions to integrate as many recommendations as possible over time to enhance local reef resilience to climate change.

These recommendations also support international initiatives focused on biodiversity and conservation targets (e.g., CBD Post‐2020 Global Biodiversity Framework; 30 × 30; UNFCCC COP 27), which are increasingly recognizing the use of restoration for achieving social and ecological outcomes. Key to supporting these global efforts is the demonstration of how countries can meet their biodiversity and climate adaptation goals through targeted coral reef restoration. Global conservation and climate change commitments are transformational opportunities to use restoration to stimulate social–ecological recovery, and the strategic integration of resilience and climate change adaptation into restoration practices in the coming decade is likely to be crucial to this effort. This work provides a first opportunity to address the gap in implementation of restoration to promote reef resilience and climate adaptation and seeks to assist coral reef managers and restoration practitioners to deliver on local and global commitments to sustain coral reefs in the coming decades. While the future of coral reefs is critically dependent on the strongest possible global reductions in greenhouse gas emissions and climate change mitigation, resilience‐based coral reef restoration plays an essential role in maintaining these valuable ecosystems while global climate action is achieved.

## Data Availability

Data sharing is not applicable to this article as no new data were created or analyzed in this study.
